# Susceptibility and immunity to helminth parasites

**DOI:** 10.1016/j.coi.2012.06.003

**Published:** 2012-08

**Authors:** Rick M Maizels, James P Hewitson, Katherine A Smith

**Affiliations:** Institute of Immunology and Infection Research, University of Edinburgh, UK

## Abstract

Parasitic helminth infection remains a global health problem, whilst the ability of worms to manipulate and dampen the host immune system is attracting interest in the fields of allergy and autoimmunity. Much progress has been made in the last two years in determining the cells and cytokines involved in induction of Type 2 immunity, which is generally protective against helminth infection. Innate cells respond to ‘alarmin’ cytokines (IL-25, IL-33, TSLP) by producing IL-4, IL-5 and IL-13, and this sets the stage for a more potent subsequent adaptive Th2 response. CD4^+^ Th2 cells then drive a suite of type 2 anti-parasite mechanisms, including class-switched antibodies, activated leukocytes and innate defence molecules; the concerted effects of these multiple pathways disable, degrade and dislodge parasites, leading to their destruction or expulsion.

## Highlights

► New studies identify innate lymphoid cells initiating immunity to helminths. ► The role of epithelial cells in both detection and expulsion of parasites. ► The concerted mechanisms that protect us from infection. ► The role of regulatory T cells in modulating protection. ► New immunoregulatory populations including macrophages, DCs and B cells.

**Current Opinion in Immunology** 2012, **24**:459–466

This review comes from a themed issue on **Host pathogens**

Edited by **Anne O’Garra** and **Eric Pamer**

For a complete overview see the Issue and the Editorial

Available online 12th July 2012

0952-7915/$ – see front matter, © 2012 Elsevier Ltd. All rights reserved.

**http://dx.doi.org/10.1016/j.coi.2012.06.003**

## Introduction

The immune system maintains our defences against both microparasites (viruses and bacteria) and macroparasites (single-celled protozoa and multicellular metazoa). While we have an increasingly comprehensive understanding of anti-microbial mechanisms, we have still to reach an equivalent analysis of immunity to extracellular parasites – mostly but not exclusively helminth worms (Box 1). Helminth parasites are generally well-adapted to their definitive hosts, yet closely related host species, and certain genotypes of their normal host organism, can be resistant to infection. The finely attuned rules that govern susceptibility and resistance are now becoming clearer with the definition of both innate and adaptive mechanisms for countering helminth infection.

Over the past 2 years, new studies have uncovered the intricacy and multiplicity of immune defences against helminths, in particular the integral role of innate immune cells both as inducers and effectors at every stage of infection [1,2]. Innate cells have been recognised as major contributors of the cytokines (IL-4, IL-5 and IL-13) that drive the canonical anti-helminth type 2 response [3,4]. Adaptive immunity encompasses the Th2 effector cell population, as well as regulatory T cell populations that minimise pathology but can block expulsion of parasites [5]. The ambivalent role of other immune cell types is seen among B cells that can act to both promote [6] or impede [7,8] immunity, as well as dendritic cells and macrophages as detailed below. The induction of these immunosuppressive populations can account for the ability of helminth infections to dampen allergies and other immunopathologies, an effect colloquially termed the ‘hygiene hypothesis’ [9,10].

Resistance to infection and immune clearance of helminths do not rest so much on one particular cell phenotype or a single molecular mechanism of killing, but rather the orchestration of multiple pathways that disable, degrade and dislocate parasites leading to their expulsion. However, regulatory pathways that either develop intrinsically to reduce pathology, or result from parasite immune evasion strategies, can block successful immunity and render the host susceptible. We discuss below the components and factors recently identified in this fascinating set of interactions.

## What drives Th2 immunity to helminths?

Since the original Th1/Th2 dichotomy emerged, it has become clear that in most instances resistance to infection requires a concerted Th2 response, dependent upon the nexus of IL-4Rα-mediated signalling [2,11]. Recent work has highlighted the role of innate cell populations in triggering this response: following infection, production of ‘alarmin’ cytokines (IL-25, IL-33 and TSLP) by epithelial cells stimulates innate lymphoid cells (ILCs, also termed nuocytes) to produce type 2 cytokines, in particular IL-5 and IL-13 [12^••^,13^••^,14^••^]. Notably, mice deficient in IL-25 (IL-17BR^−/−^) or IL-33 (T1/ST2) receptors mount poor type 2 responses and are more susceptible to infection with *Nippostrongylus brasiliensis*, while immunity can be restored to these animals by transfer of ILCs acting in an IL-13-dependent manner [13^••^]. Interestingly, nuocytes can be considered type 2 ILCs requiring the transcription factor RORα [15], distinguishing them from RORγt-dependent IL-17A-producing and IL-22-producing ILCs that initiate the pathogenesis of inflammatory bowel disease [16]; however both populations rely on the expression of the Id2 transcriptional repressor for their differentiation [16].

In the lung, IL-33 production by epithelial cells was also shown to stimulate innate helper cell IL-5 production, resulting in eosinophilia and a contribution to worm expulsion in mice infected with *Strongyloides venezuelensis* [17]. IL-33 release is itself dependent on prior production of trefoil factor 2 (TFF2), a central player in wound healing, during migration through the lung by *N. brasiliensis* larvae [18^••^] and TFF-2-deficient mice consequently showed impaired Th2 responsiveness and worm expulsion. TFF-2 is also highly expressed in the mucosa of sheep resistant to similar intestinal nematode infection [19]. In parallel with, and independent of, the TFF2-IL-33 axis, epithelial cells also produce TSLP when perturbed by infection, affecting a range of innate targets, most notably dendritic cells and basophils [20].

While the adaptive Th2 response is closely linked to IL-4 production, ILCs were not found to produce this cytokine [21^••^], indicating that innate IL-13 may be sufficient for signalling naive T cells through the IL-4R. By contrast, IL-4^+^ basophils expand rapidly following *N. brasiliensis* infection [22], and it was reported that these innate cells were necessary and sufficient for Th2 immunity to *T. muris* [23]. More extensive investigations have not supported the latter contention, for example, in *N. brasiliensis* [24], *S. mansoni* [25] and *H. polygyrus* [26] infections. By contrast, basophil depletion has little effect on initial Th2 induction to *N. brasiliensis* or *S. mansoni* [24–26,27^•^], and basophil-derived IL-4 found to be required for *N. brasiliensis* expulsion only when CD4^+^ T cells were unable to produce this cytokine [28]. Hence in this model innate and adaptive IL-4 act redundantly. Similarly in *L. sigmodontis*, basophils act are non-essential amplifiers of Th2 [29].

Studies with IL-4 and IL-13 reporter mice have revealed the compartmentalisation of adaptive and innate cell populations mediating immune responsiveness to *N. brasiliensis*. Whereas IL-13-producing CD4^+^ T cells and ILCs are located in the tissue and regulate worm expulsion and eosinophilia, IL-4 producing CD4^+^ T-follicular helper cells (TFH) are concentrated in draining lymph node and drive the humoral response following *N. brasiliensis* infection [30].

One important consequence of the dominant Th2 response in the lung is that it promotes wound healing; whereas production of IL-17 was found to initially contribute to acute inflammation following *N. brasiliensis* infection, subsequent IL-4Rα signalling was required to prevent haemolysis and pathology [31]. The common strands between the response to tissue damage and to helminth infection may signal an evolutionary link in the very origin of Th2 mechanisms, as well as shared mechanisms to defend and repair tissues invaded by parasites [2,32].

**Figure 1:**
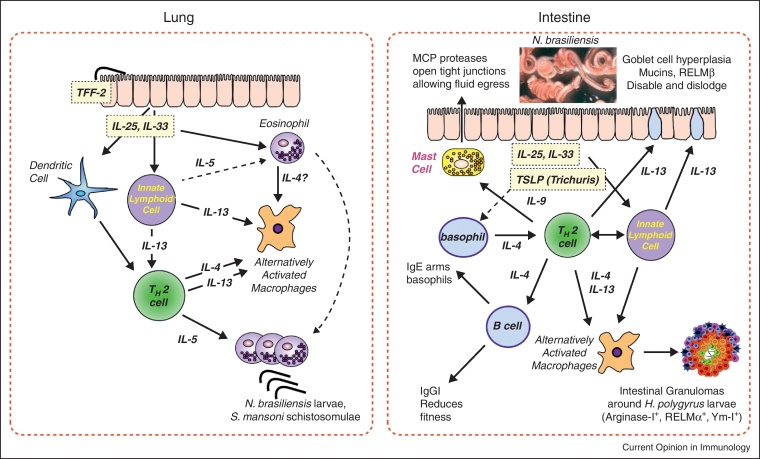
Innate and adaptive immune mechanisms in helminth infections in the lung and gut. In the lung, traversing parasites elicit epithelial cell alarmin production (IL-25 and IL-33, the latter requiring preceding expression of TFF-2) that activate innate cells including innate lymphoid cells, eosinophils and DCs; DC stimulation of helminth-specific Th2 results in amplified production of IL-4, IL-5 and IL-13 that drive alternative activation of macrophages and considerable expansion of eosinophils, implicated in immune attack on migrating helminth larvae. In the intestine, similar innate triggers (including TSLP) generate innate and adaptive populations, including mast cells and basophils, and act on intestinal epithelial cells to induce goblet cell differentiation and expression of molecules inimical to parasite persistence. Adaptive Th2 response promotes B cell production of protective antibodies.

The new paradigm of the tissues raising the first alarm of helminth infection, through innate cytokines, is an attractive one; but even innate helper cells require continuing stimulation by RAG-dependent B or T cells [13^••^], and the adaptive Th2 response also requires classical MHC class-II-mediated antigen presentation by dendritic cells [25], so that any new model of helminth immunity must stress the co-operation and interdependence of the innate and adaptive arms [2]. Thus, while innate cells sound the alarm on helminth encounter, effective immunity demands activation of the T cell compartment. Through this bridge to adaptive immunity, a suite of type 2 innate effector mechanisms come into play, including alternative activation of macrophages, recruitment of eosinophils, and transformation of the intestinal environment through mast cell, goblet cell and epithelial cell activation (Figure 1).

## A 3D view of anti-helminth immunity: disable, degrade and dislodge

Despite their essential role in driving protective immunity to helminths, T cells cannot directly damage the parasites – they remain armchair generals conducting attacks through remote means; many of the effector pathways are ancient innate defence mechanisms involving epithelial cells or other tissue populations. In concert these mechanisms achieve three effects:

*Disabling* effects are those that restrict parasites’ growth and motility, and reduce their overall fitness and ability to reproduce. These effects may be mediated through antibodies neutralising key physiological functions [6,33–35], together with innate defensin-like molecules such as RELM-β that are postulated to confound sensory inputs [36]. Amino acid deprivation through (for example) macrophage-expressed arginase may also retard larval development. An important consequence of attenuating the fitness and fecundity of helminths in this fashion is the mitigation of disease where for example eggs or migrating larvae provoke pathology.

*Degrading* effects are represented by cumulative damage to parasite integrity, for example through granulocyte attack, which may be guided by specific antibodies and/or amplified by complement components and other serum factors. In tissue infections, progressive calcification of adult worms can occur, depriving parasites of surface nutrient exchange. In this respect, it can be noted that while no single cell subset is responsible for this type of attack, a number of transgenic overexpression models report that elevated mast cell [37] or eosinophil [38] responses are able to exert strong anti-parasite effects, while nitric oxide from macrophages and neutrophils can harm tissue larvae [39]. Hence, in physiological settings it is likely that multiple cell types contribute incrementally to damaging parasites.

*Dislodging* parasites, in particular those in the gastrointestinal tract, is achieved by making the chosen niche untenable. IL-25 and IL-13 play a critical role here; promoting changes in intestinal function following *N. brasiliensis* infection [40] as well as the more rapid cell turnover (the ‘epithelial escalator’) in *T. muris* [41]. IL-13 also induces a switch in intestinal mucins from the dominant Muc2 to a form not normally expressed in the intestine, Muc5ac, which is required for normal expulsion of both these parasites [42^•^]. Likewise, the increase in epithelial permeability during *H. polygyrus* infection, increasing fluid transfer, is STAT-6-dependent [43]. Furthermore, IL-4Rα-mediated signalling activates smooth muscle cells, leading to hypercontractibility during infection, while also stimulating Th2-promoting cytokine production from muscle cells [44].

Interestingly, not all helminths can be subjected to these measures: for example cysts of the tapeworm *Echinococcus granulosus* form in liver and other tissues, which once established cannot be eliminated by the immune system [45]. In another exceptional instance, immunity to the blood-borne microfilarial stage of *B. malayi* appears to be mediated by Th1 cells and inhibited by IL-10 [46].

Two further exceptions illustrate the complexity of helminth-immune interactions at the single cell type level: eosinophils promote infection with *Trichinella spiralis* [39] while mast cell degranulation during filarial larval invasion of the skin may facilitate infection by raising vascular permeability; CCL17^−/−^ mice fail to inhibit local mast cell responses and are more susceptible [47].

## Tregs – the key to susceptibility?

**Figure 2:**
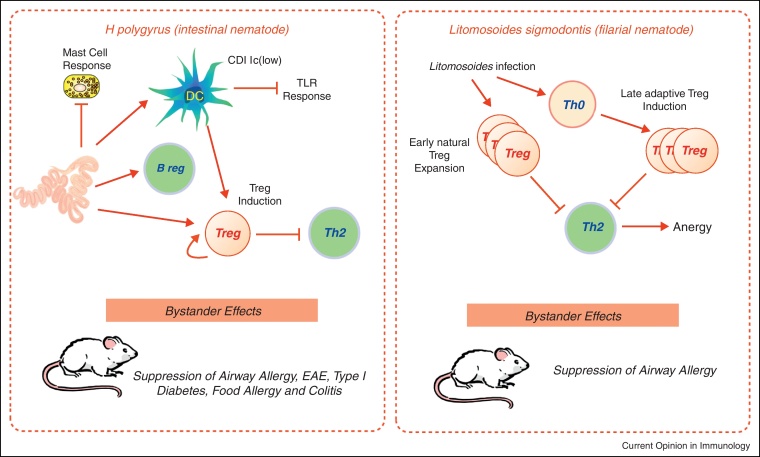
Two models of immunoregulation in helminth infection are *H. polygyrus* and *L. sigmodontis*. In *H. polygyrus*, infection suppresses mast cell hyperplasia and modulates DC responsiveness to TLR ligation; in addition both Tregs and Bregs are generated and Th2 responses suppressed. As well as parasite-specific immunity, bystander responses to allergens and autoantigens are also suppressed. In *L. sigmodontis*, it is thought that natural T regs expand early in infection, followed by a later wave of adaptive Tregs; these populations induce anergy in effector T cells, impairing both parasite immunity and allergic responses to third-party allergens [5].

Successful helminth infection generally requires downregulation of Th2 responsiveness, which is evident in the chronic phases of most human and murine helminth infections [5] as summarised in Figure 2. In mouse models, Th2 immunity can be rescued by interference with Treg function through direct deletion of Foxp3-expressing cells [48], administration of antibodies to CD25, CTLA-4 and GITR [5] or inhibition of TGF-β signalling [49]. However, in other systems protective immunity is not restored by the first of these means [50], suggesting that multiple Th2-inhibiting mechanisms may be invoked during helminth infection to dampen protective immunity.

Evidence for Tregs in humans is strengthening with recent reports that filarial and schistosome patients have higher frequencies of Foxp3^+^ cells [51^•^,52], and that *in vitro* depletion of CD25^+^ PBMC from geohelminth-infected patients increases immune responsiveness even to bystander antigens [53]. In humans, chronic helminth infection is also associated with higher IL-10 production, for example in children harboring gastrointestinal nematodes [54]. Interestingly, anti-IL-10 treatment during praziquantel treatment of schistosome-infected mice leads to the development of protective immunity that otherwise is not seen; hence, IL-10 plays a role in chronic infection of impairing immunity [55]. Since the primary source of IL-10 in most helminth infections is the CD4^+^Foxp3^−^ subset, it will also be interesting to identify whether schistosome immunity in this system is directly impeded by classical Tregs.

The expanded profile of Tregs in helminth infections [56] may result from activation of pre-existing (natural or thymic) Tregs or the *de novo* induction of Tregs from naïve peripheral Th0 precursors. *In vivo* labelling studies demonstrate early expansion of natural Tregs in *L. sigmodontis* infection [57], while in *H. polygyrus*-infected mice, conversion of naïve ovalbumin-specific T cells to Foxp3^+^ Tregs was amplified [49]. At the molecular level, *H. polygyrus* was found to secrete a TGF-β-like factor that directly induced Foxp3 expression while the ability of Schistosome egg antigen to drive the same effect was mediated through DCs exposed to the major product ω-1 [58]. A novel phenotype of DC has also been found in the lymph nodes draining the site of *H. polygyrus* infection, a CD11c^low^CD103^−^ cells subset that expands during infection and preferentially induces Foxp3 expression in naive T cells [26].

The net effect of Treg expansion in helminthiases may be to protect the host against more extreme pathological outcomes of infection. Thus, anti-CD25 or anti-GITR led to increased intestinal pathology in *T. muris* infection and in the case of anti-GITR faster worm expulsion [59]. The deletion of Foxp3^+^ Tregs in DEREG mice resulted in aggravated small intestinal epithelial cell dysfunction in *H. polygyrus* infection [50]. Similarly, colonic inflammation due to schistosome egg exposure provokes granuloma formation, which over time is infiltrated by Tregs and downmodulated in intensity; depletion of CD4^+^CD25^+^ Tregs abolishes downmodulation while reversing effector cell anergy *in vivo* [60].

One relative newcomer to the regulatory spectrum is the regulatory B cell, now implicated in several helminth systems. For example, mesenteric lymph node B cells from *H. polygyrus*-infected mice were able to suppress both airway allergy and experimental autoimmune encephalomyelits in uninfected recipients in an IL-10-independent manner [8]. By contrast, IL-10 was instrumental in the ability of splenic B regs from *S. mansoni* infected mice to suppress allergy [7,61], and inhibition correlated with recruitment of Foxp3^+^ Tregs *in vivo*. Notably, elevated numbers of Breg phenotype (IL-10^+^CD1d^hi^) cells were also found in human schistosomiasis patients [61].

## Alternatively activated macrophages – immunologists or physiologists?

Helminth infections provide the canonical setting for an important phenotype of Alternatively Activated Macrophage (AAM), which are stimulated by high levels of IL-4 and IL-13 [2]. In serous cavities, helminth infections drive the proliferation of resident macrophages without recruitment of blood-derived monocyte populations in an IL-4Rα-dependent manner [62]. Similar phenotype cells accumulate in the intestinal tissue during gut nematode infection, and in the case of *H. polygyrus*, immunity of drug-cured mice to challenge infection is ablated following depletion of these macrophages [63].

AAM phenotype cells are also involved in fundamental homeostatic and physiological balances. For example, following *N. brasiliensis* infection in the lung, AAMs are essential to repair the tissue damage [31], while in the same infection stimulation of AAMs in adipose tissue results in improved glucose tolerance in metabolic disturbance [64]. Counterbalancing these benevolent effects, it should be noted that the induction of type 2 phenotype of AAMs by *N. brasiliensis* results in compromised resistance to *Mycobacterium tuberculosis* in the lung [65].

## Conclusion

Helminth parasites are still, even in the 21st Century, remarkably prevalent across the world, and in historical time would have been near-ubiquitous. Inevitably then, helminths have made an enormous imprint on the design and function of the mammalian immune system, not least in the evolution of the Type 2 response. As argued elsewhere [2], the imperative to exclude parasites while minimising collateral pathology has led in different ways to two of the most striking features of infection: the redundancy and parallelism between innate and adaptive effector responses, and the prominence of regulatory populations and mechanisms to focus and restrain host immunity. Recent rapid progress in defining these interactions is both completing our understanding of the full functional range of the immune system, and suggesting innovative new strategies for the control of helminth parasite infections.

## References and recommended reading

Papers of particular interest, published within the period of review, have been highlighted as:

• of special interest
•• of outstanding interest
